# Understanding the effects of platelet transfusions in neonates

**DOI:** 10.1038/s41390-025-04628-3

**Published:** 2025-11-15

**Authors:** Robert W. Maitta

**Affiliations:** Department of Pathology, University Hospitals Cleveland Medical Center, Cleveland Ohio and Case Western Reserve University School of Medicine, Cleveland, OH USA

## Abstract

Platelet transfusions represent a common life-saving intervention used in intensive critical settings to treat neonates with thrombocytopenia, at risk of or actively bleeding, or undergoing clinical complications that make transfusions part of the management. Even though it is intended to help restore hemostasis in patients, platelet transfusions can, under certain circumstances, lead to negative effects, greater inflammation, and rarely increased mortality. It is the occurrence of these unforeseen and yet to be defined transfusion-associated complications that are at the center of a renewed effort to identify those factors increasing risk or that make a subset of patients more likely to develop adverse events as a result of transfusions. Platelets are active members of the native immune system and, as such, express surface receptors that mediate directly cross-talk with immune cells or do so indirectly though secretion of cytokines and chemokines that activate immune cells. Together with inherent differences between donated adult platelets and platelets in neonates, the reasons for negative responses begin to come into focus.

Commentary on Davenport et al. Effects of Platelet Transfusions on Neonatal Bleeding and Inflammation. Pediatr Res. 2025.^1^

Neonatal platelet transfusions represent one of the most common approaches in clinical practice used to treat patients either born with low counts or who develop thrombocytopenia as a result of complicated stays in neonatal intensive care units, such as during infections. At times, however, these transfusions can have undesired and unexpected deleterious effects that make a better understanding of how transfusions affect a neonate an area that requires further scrutiny. Indeed, results from randomized clinical trials have indicated that transfusions of neonates and preterm patients at counts of <50 × 10^9^/L resulted in greater mortality compared to transfusions at counts <25 × 10^9^/L.^2,3^ Likewise, a meta-analysis looking at platelet transfusions in preterm infants found that, in addition to a higher mortality risk, patients were also more likely to develop sepsis, necrotizing enterocolitis, and even paradoxically intraventricular hemorrhage as a result of transfusion.^4^

The reasons for adverse events resulting from platelet transfusions are likely multifactorial and linked to inherent factors associated with platelet units and recipients themselves. Data indicating that platelet transfusions lead to immune and inflammatory changes in recipients that are either mediated directly by platelets or by their active secretion of mediators such as cytokines present in transfused units have been reported.^5^ For example, CXCL5, CD40, and TGF-β have been found increased after platelet transfusions in premature infants, resulting in exacerbation of inflammatory effects over lung, white matter, and worsening necrotizing enterocolitis.^6^ It is possible that platelet dosage could play a role in the occurrence of these adverse events, especially if patients are given a higher volume of platelet aliquots in relation to their body weight, thus exposing them to higher concentrations of cytokines and/or platelets, leading to undesired effects.^7^ Consequently, this is something that will need to be investigated to establish if high platelet volume per Kg of body weight accentuates negative effects observed during platelet transfusions.

The immunomodulatory effects brought about by platelet transfusions can be secondary to differences between platelets from adults and immune cells in premature infants and neonates. Neonatal platelets are uniformly less reactive to agonists like thrombin and epinephrine, while adult platelets are more immune-differentiated, due to the higher expression of receptors and mediator molecules that affect the immune response of the recipient.^8^ In vitro experiments using platelets from adult donors transfused into thrombocytopenic neonatal blood resulted in shorter closure times and overall higher clot strength and firmness than similar transfusions using autologous neonatal platelets, suggesting that significant differences between neonatal and adult hemostatic systems exist, and that this developmental mismatch may explain some of the effects associated with platelet transfusions on neonatal hemostasis and immune responses.^9^ This is similar to what has been seen in transfusions of platelets from adult mice into mouse pups, which lead to an acute inflammatory and trafficking monocyte phenotype that is dependent on P-selectin expression.^8^ Furthermore, higher number of platelet transfusions increases the risk to the patient in a cumulative manner due to incremental fibrinogen binding exposure and P-selectin expression in response to agonists.^9^ Likewise, P-selectin on donated platelets is more likely to bind to neutrophil PSGL-1 and trigger release of mediators, leading to formation of neutrophil extracellular traps (NETs).^10^

This makes the report by Davenport and colleagues one important step toward understanding mechanisms by which transfused platelets affect the immunologic milieu of recipients. In this study, researchers analyzed the effects of platelet transfusions at two institutions’ neonatal intensive care units. Seventy patients were enrolled during a four-year period. Consents were obtained from parents of infants with platelet counts <100 × 10^9^/L, but they became active study participants (42 patients) once platelet count decreased to <50 × 10^9^/L or if platelet transfusion was requested. Both platelet counts and number of platelet transfusions were recorded until the platelet count was ≥75 × 10^9^/L for ≥72 h without further transfusions. In a subgroup of active patients, a blood specimen (0.5 mL) was drawn via central access or heel stick 1 h before and 2- or 4-h post platelet transfusion and processed within 1 h. The study then proceeded to run a human cytokine/chemokine comprehensive panel containing 48 analytes. These cytokines included mediators known to trigger immune activation/stimulation and inflammation. To determine if these cytokines were already present in the units prior to transfusion, samples from transfused units were collected and processed similarly to patient samples to derive supernatants to be tested for cytokines and the presence of mitochondrial DNA, the latter used as a marker of NET formation.

All platelet units transfused to infants were collected via apheresis, unwashed, the majority of which were irradiated and resuspended in autologous plasma, with the remainder being pathogen-reduced units stored in platelet additive solution. Units were stored for a mean of 4.9 days, and a mean aliquot volume (dose) of 12.0 mL/kg was given during each transfusion event. Mean mitochondrial DNA concentration in platelet supernatant of units transfused was 52.2 ng/mL. Not surprisingly, they observed that transfusions resulted in a mean platelet count increment of 15 × 10^9^/L. Notably, they did not observe changes in bleeding scores of patients from their baselines regardless if they received platelet transfusions or not.

Two hours post-transfusion, investigators observed significant increases in platelet-derived growth factor (PDGF)-α (2.2-fold, *p* = 0.018) and PDGF-β (9.3-fold, *p* = 0.046). Vascular endothelial growth factor (VEGF)-α was also 2.6-fold higher compared to pre-transfusion levels but lacked significance (*p* = 0.06). Interestingly, 4 h post-transfusion, both PDGF-α and PDGF-β remained significantly elevated (2.1-fold, *p* = 0.004 and 1.9-fold, *p* = 0.007, respectively); however, RANTES, a strong immune system regulator, exhibited the greatest increase at this time point (6.5-fold, *p* = 0.034). To further discern the origin of these increases, these four cytokines were measured in platelet supernatants of transfused units, and all were found to be in higher concentrations than pre-transfusion infant plasma. Therefore, observed PDGF-α, PDGF-β, and VEGF post-transfusion concentration increases could be secondary to the content of transfused platelets. On the other hand, the results with RANTES were significantly higher than expected (*p* = 0.01), suggesting that some of the observed RANTES is produced after transfusion by the recipient, and this higher production was associated with length of platelet storage and the concentration of mitochondrial DNA in the platelet unit. In regard to changes in NET, they found that only a patient’s low birth weight appeared to correlate with their formation. This implies that premature infants are at a greater risk than full-term neonates.

In conclusion, continuous need for donated platelets collected from an adult population indicates that potential complications of transfusions will likely continue to occur. These platelets work optimally in donors’ circulation, and their phenotypes may not always be compatible with patients, such as neonates, whose platelets are found at a different state of activation. Negative responses to platelet transfusions reported in neonates and premature infants are not traditional transfusion reactions, but represent complications due to the inherent physiologic differences between donated platelets and neonatal circulation. Thus, for neonates and preterm infants with a developing immune system and having a low birth weight, repeated platelet transfusions may increase overall risk of greater RANTES, which results in worsening inflammation and greater NET formation, resulting in tissue damage and enhanced inflammatory responses. Likewise, differences between adult and neonatal platelets in their immune function capabilities and gene expression provide a cohesive mechanistic explanation for the observed higher inflammation, bleeding, and neurodevelopmental complications, and reports of higher mortality and harm in neonate and preterm infants.
